# Adverse Events during Bowel Preparation and Colonoscopy in Patients with Acute Lower Gastrointestinal Bleeding Compared with Elective Non-Gastrointestinal Bleeding

**DOI:** 10.1371/journal.pone.0138000

**Published:** 2015-09-14

**Authors:** Ryota Niikura, Naoyoshi Nagata, Takuro Shimbo, Toshiyuki Sakurai, Tomonori Aoki, Shiori Moriyasu, Katsunori Sekine, Hidetaka Okubo, Kazuhiro Watanabe, Chizu Yokoi, Atsuo Yamada, Yoshihiro Hirata, Kazuhiko Koike, Junichi Akiyama, Naomi Uemura

**Affiliations:** 1 Department of Gastroenterology and Hepatology, National Center for Global Health and Medicine, Tokyo, Japan; 2 Department of Gastroenterology, Graduate School of Medicine, The University of Tokyo, Tokyo, Japan; 3 Ohta Nishinouchi Hospital, Koriyama, Japan; 4 Department of Gastroenterology and Hepatology, National Center for Global Health and Medicine, Kohnodai Hospital, Chiba, Japan; University Hospital Llandough, UNITED KINGDOM

## Abstract

**Background:**

There are limited data on the safety of colonoscopy in patients with lower gastrointestinal bleeding (LGIB). We examined the various adverse events associated with colonoscopy in acute LGIB compared with non-GIB patients.

**Methods:**

Emergency hospitalized LGIB patients (n = 161) and age- and gender-matched non-GIB controls (n = 161) were selected. Primary outcomes were any adverse events during preparation and colonoscopy procedure. Secondary outcomes were five bowel preparation-related adverse events–hypotension, systolic blood pressure <100 mmHg, volume overload, vomiting, aspiration pneumonia and loss of consciousness–and four colonoscopy-related adverse events–including hypotension, perforation, cerebrocardiovascular events and sepsis.

**Results:**

During bowel preparation, 16 (9%) LGIB patients experienced an adverse event. None of the LGIB patients experienced volume overload, aspiration pneumonia or loss of consciousness; however, 12 (7%) had hypotension and 4 (2%) vomited. There were no significant differences in the five bowel preparation-related adverse events between LGIB and non-GIB patients. During colonoscopy, 25 (15%) LGIB patients experienced an adverse event. None LGIB patient had perforation or sepsis; however, 23 (14%) had hypotension and 2 (1%) experienced a cerebrocardiovascular event. There was no significant difference in the four colonoscopy-related adverse events between LGIB and non-GIB patients. In addition, no significant difference in any of the nine adverse events was found among subgroups: patients aged ≥65 years, those with comorbidities, and those with antithrombotic drug use.

**Conclusions:**

Adverse events in bowel preparation and colonoscopy among acute LGIB patients were low. No significant difference was found in adverse events between LGIB and non-GIB patients. These adverse events were also low in elderly LGIB patients, as well as in those with co-morbidities and antithrombotic drug use, suggesting that colonoscopy performed during acute LGIB did not increase adverse events.

## Introduction

Colonoscopy is an essential tool for optimal management of acute lower gastrointestinal bleeding (LGIB), as it can identify the bleeding source and indicate therapeutic modalities [1]. A higher risk of colonoscopy-related adverse events may occur in elderly patients with co-morbidities or in patients using antithrombotic drugs when urgent or intervention colonoscopy is necessary [2,3]. However, the safety of bowel preparation and colonoscopic interventions remains unknown.

Bowel preparation improves visualization of the colon [3] but requires oral administration of polyethylene glycol (PEG)–based solutions. In acute LGIB settings, bowel preparation potentially increases the risk of vomiting, aspiration pneumonia, volume overload, and a change in vital signs with blood loss [1]. In addition, perforation, cardiovascular events and sepsis have been reported as colonoscopic-related adverse events [4].

In this study, we investigated various adverse events and hemodynamic instability during bowel preparation and colonoscopy in emergently hospitalized patients with acute LGIB. The specific objectives of this study were 1) to elucidate the adverse event rates during bowel preparation and colonoscopy in acute LGIB and non-GIB patients and 2) to compare these adverse events rates according to age, gender, co-morbidities and antithrombotic drug use.

## Materials and Methods

### Study design, participants, and setting

**Fig 1:**
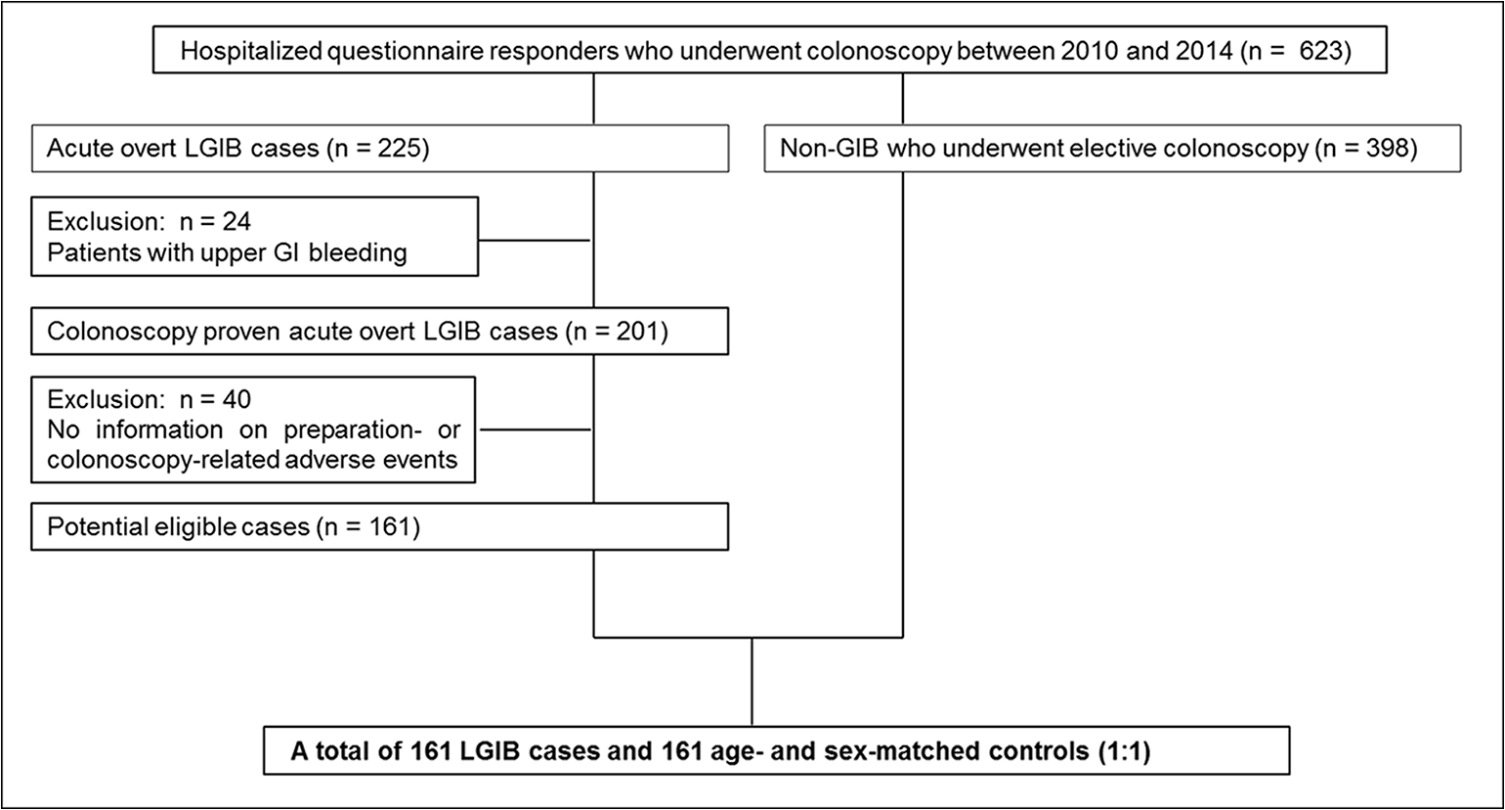
Study flow chart.

This was a retrospective review of 623 hospitalized Japanese adults who underwent colonoscopy and completed a questionnaire in a prospectively recorded database [5–8]. Details regarding the patient population and data collection are outlined in a previous publication [5–8]. The study was conducted at the Department of Gastroenterology and Hepatology of the National Center for Global Health and Medicine (NCGM) between September 2009 and June 2013. The NCGM is one of the largest emergency hospitals in Tokyo, Japan. The LGIB and non-GIB patient criteria are described in Fig 1.

In the LGIB cases, the inclusion criteria were age >18 years and outpatients with acute onset of overt frequent, massive, continuous LGIB who required urgent hospitalization [7]. We excluded patients with evidence of a source of bleeding on upper endoscopy (n = 24), and those patients who lacked vital sign data during preparation and colonoscopy, and adverse event data relative to preparation or colonoscopy (n = 40). After applying these criteria, a total of 161 patients with acute LGIB were eligible (Fig 1).

Among the non-GIB cases, the inclusion criteria were age >18 years, no evidence of overt GI bleeding, and hospitalization for colorectal cancer screening, post-polypectomy surveillance or examination of GI symptoms due to an advanced age, severe co-morbidities, live in a distant place, or patient illness. In total, 398 patients without any evidence of GI bleeding who underwent elective colonoscopy were eligible (Fig 1).

To minimize confounding effects, controls were selected randomly among individuals matched for decennial age and gender at a ratio of 1:1. The final analysis included data from 322 subjects (161 LGIB patients and 161 non-GIB patients; Fig 1). This study was conducted according to the principles of the Declaration of Helsinki, and approved by the ethics committee of NCGM (No. 1579). Patient information was anonymized and deidentified prior to analysis, and the need for patient consent was waived.

### Acute LGIB criteria and treatment of LGIB

All colonoscopies were performed using an electronic video endoscope (Olympus Optical, Tokyo, Japan) after full bowel preparation. An enema was performed in patients who had not completely consumed the polyethylene glycol solution, and a water-jet device (Olympus Flushing Pump; Olympus Optical, Tokyo, Japan) was used to obtain better visualization in cases of inadequate preparation [9]. Overt LGIB originating in the large or small bowel was diagnosed by endoscopy. Briefly, large bowel hemorrhage included frank melena or rectal bleeding with no evidence of a source on upper endoscopy with nasogastric aspirate, capsule endoscopy, or double-balloon endoscopy. We made definitive diverticular bleeding diagnoses based on colonoscopic visualization of diverticula exhibiting the stigmata of recent hemorrhage (such as active bleeding, a visible vessel or an adherent clot) [4,10]. We made presumptive diverticular bleeding diagnoses based on colonoscopic visualization of fresh blood localized to the colonic diverticula when a potential bleeding source was evident by total colonoscopy.[4,10] Small bowel bleeding was diagnosed by capsule endoscopy or double-balloon endoscopy. Overt LGIB of unknown origin or hemorrhoidal bleeding was defined as a clinically significant drop in hematocrit ≥10% and/or hemoglobin ≥2 g/dL from baseline levels according to previous criteria [11]. Patients who did not meet these criteria were excluded. We performed hemostatic interventions based on an algorithm [3], and endoscopic treatment using clipping or ligation was the first-line therapy in patients with LGIB. Interventional radiology was reserved for patients with severe bleeding who failed endoscopic treatment. Surgery was reserved for patients with persistent bleeding who failed interventional radiology.

### Data sources and measurement

A questionnaire was completed by medical staff during a face-to-face interview with each patient at the endoscopy unit prior to colonoscopy. The questionnaire collected information regarding alcohol consumption, smoking, use of medications such as non-steroidal anti-inflammatory drugs (NSAIDs), low-dose aspirin, non-aspirin antiplatelet drugs, and anticoagulants [5–8]. Antithrombotic drugs included low-dose aspirin, non-aspirin antiplatelet drugs, and anticoagulants. For medications, patients were asked to indicate which drugs, if any, they had used, based on the pictures of drugs on the questionnaire [5–8]. Use of a drug was defined as intermittent or regular oral administration within 1 month before the interview. Body mass index (BMI) and information regarding 16 co-morbidities were collected from an electronic endoscopic database (Solemio Endo; Olympus Medical Systems, Tokyo, Japan) [5–8] or electronic medical records. Comorbidity was calculated according to the Charlson comorbidity index, which is a classifying prognostic comorbidity index [12] that has been validated extensively for GI bleeding [13].

### Adverse events and vital signs during bowel preparation and colonoscopic procedures

The primary outcome was any adverse event during bowel preparation and the colonoscopy procedure. Secondary outcomes were five bowel preparation-related adverse events–hypotension, systolic blood pressure <100 mmHg, volume overload, vomiting, aspiration pneumonia and loss of consciousness,–and four colonoscopy-related adverse events–hypotension, perforation, cerebrocardiovascular events, and sepsis.

Nursing staff monitored vital signs regularly. Blood pressure was measured at least twice a day during hospitalization at rest in the sitting position by nursing staff, and these data were recorded in electronic medical records during hospitalization. When vital sign changes were seen during preparation, the nursing staff consulted the attending physician, who diagnosed adverse events using laboratory testing and radiographic examination by x-rays, computed tomography or ultrasound echocardiography. Colonoscopy-related adverse events were defined as events within 24 h after the colonoscopy. Cerebrocardiovascular events included angina pectoris, myocardial infarction and cerebrovascular infarction.

### Statistics

Patient characteristics were compared between the LGIB and non-GIB cases using a X^2^ test or Fisher’s exact test as appropriate. Continuous variables, such as BMI and Charlson comorbidity index, were compared using the Mann-Whitney U test. We used conditional logistic regression analysis to confirm whether various adverse events were associated with LGIB. In multivariate analysis, we adjusted for potential confounding factors significantly associated with LGIB in univariate analysis. The crude and adjusted odds ratio (OR) and 95% confidence intervals (CI) were calculated. To calculate the power to detect a difference of preparation-related adverse event rate in LGIB vs. non-GIB, we performed a post hoc power calculation.

In subgroup analyses of LGIB patients, we evaluated adverse events between groups according to age ≥65 years, Charlson comorbidity index ≥2, past history or presence of congestive heart failure (CHF), and chronic kidney disease (CKD) with an estimated glomerular filtration rate <60 mL/min/1.73m^2^, and antithrombotic drug use. The threshold of statistical significance was P <0.05. All statistical analyses were performed using the STATA software (ver. 13; StataCorp, College Station, TX, USA).

## Results

### Patient characteristics

The patient characteristics are shown in Table 1. The number of patients who were unable to take the polyethylene glycol solution was 33 (20.5%) in the LGIB group and 18 (11.2%) in the non-GIB group. Of the LGIB subjects, 60% underwent colonoscopy within 24 h after admission, and the other LGIB subjects underwent colonoscopies between 2 and 5 days after admission. All of the non-GIB subjects underwent colonoscopies within 24 h after admission. The source of bleeding varied among patients, with 78 (48%) experiencing colonic diverticular bleeding and 29 (18%) experiencing bleeding due to ischemic colitis (S1 Table). After adjusting for age and gender among non-GIB patients, factors such as low-dose aspirin, non-aspirin antiplatelet use, and current alcohol use were associated with LGIB, while a high comorbidity index was associated with non-GIB patients. There were 25 (16%) LGIB patients who required a transfusion within 24 h of admission.

**Table 1:** Baseline characteristics of the study participants at admission (n = 322).

	LGIB (n = 161)	Non-GIB (n = 161)	P
Mean age, (years)	65 ± 16	65 ± 16	0.93
≥ 65 years	96 (60)	97 (60)	0.91
Gender, male	92 (57)	92 (57)	1.00
Mean BMI, (kg/m^2^)	22.8, 3.4	22.7, 3.2	0.72
Current alcohol users	85 (53)	66 (42)	0.04
Current smokers	35 (22)	37 (23)	0.72
Medication†			
NSAIDs	27 (17)	18 (11)	0.15
Antithrombotic drugs	51 (32)	28 (18)	<0.01
Low-dose aspirin	33 (21)	15 (10)	<0.01
Non-aspirin antiplatelets	24 (15)	9 (6)	<0.01
Anticoagulants	11 (7)	8 (5)	0.49
Comorbidities			
Mean Charlson comorbidity index	1.7 ± 2.1	2.7 ± 2.7	<0.01
Diabetes mellitus	19 (12)	33 (21)	0.03
Diabetes mellitus with end organ damage	4 (2)	9 (6)	0.16
Hemiplegia	3 (2)	2 (1)	1.00
Cerebrovascular event	24 (15)	6 (4)	<0.01
Chronic pulmonary disease	6 (4)	4 (2)	0.75
Dementia	3 (2)	4 (2)	1.00
Connective tissue disease	6 (4)	21 (13)	<0.01
Myocardial infarct	20 (12)	16 (10)	0.48
Congestive heart failure	12 (7)	3 (2)	0.03
Ulcer disease	8 (5)	15 (9)	0.13
Renal disease (moderate or severe)	45 (28)	37 (23)	0.31
Peripheral vascular disease	7 (4)	2 (1)	0.17
Leukemia	0	1 (1)	1.00
AIDS	1 (1)	18 (11)	<0.01
Any tumor	11 (7)	29 (18)	<0.01
Metastasis solid tumor	3 (2)	5 (3)	0.72
Liver disease (mild)	8 (5)	15 (9)	0.13
Liver disease (moderate or severe)	5 (3)	1 (1)	0.21
Lymphoma	2 (1)	10 (6)	0.02

Values are presented as means ± SD or as the number of patients (%). Bolded values indicate statistical significance.

† Use of a drug was defined as regular oral administration within 1 month before the interview.

AIDS, acquired immune deficiency syndrome, BMI, body mass index, GIB, gastrointestinal bleeding, LGIB, lower gastrointestinal bleeding, NSAIDs, non-steroidal anti-inflammatory drugs, SD, standard deviation

### Bowel preparation-related adverse events

The bowel preparation-related adverse events in LGIB and non-GIB patients are shown in Table 2. During bowel preparation, 16 (9%) LGIB patients experienced an adverse event. None of the LGIB patients had volume overload, aspiration pneumonia, or loss of consciousness, although 12 (7%) experienced hypotension and 4 (2%) vomited. There were no significant differences observed among the five bowel preparation-related adverse events rates between LGIB and non-GIB patients, even after adjusting for confounding factors.

**Table 2:** Preparation-related adverse events (n = 322).

	LGIB (n = 161)	Non-GIB (n = 161)	P	Crude OR (95% CI)	Adjusted OR* (95% CI)	P
Any	16 (9)	22 (14)	0.30	0.7 (0.32–1.38)	0.5 (0.21–1.27)	0.15
Hypotension	12 (7)	19 (12)	0.19	0.6 (0.27–1.28)	0.4 (0.12–1.13)	0.08
Volume overload	0	0	NA	NA	NA	NA
Vomiting	4 (2)	3 (2)	0.70	1.3 (0.30–5.96)	2.0 (0.18–22.1)	0.57
Aspiration pneumonia	0	0	NA	NA	NA	NA
Loss of consciousness	0	0	NA	NA	NA	NA

Values in parentheses are percentages. Hypotension was diagnosed as a systolic blood pressure < 100 mmHg.

*The adjusted OR value took into account current alcohol use, antithrombotic drug use and the Charlson comorbidity index.

CI, confidence interval, GIB, gastrointestinal bleeding, LGIB, lower gastrointestinal bleeding, NA, not applicable, OR, odds ratio.

### Colonoscopy-related adverse events

The colonoscopy-related adverse events between LGIB and non-GIB patients are shown in Table 3. During colonoscopy, 25 (15%) LGIB patients experienced an adverse event. None of the LGIB patients had perforation or sepsis, although 23 (14%) had hypotension and 2 (1%) experienced cerebrocardiovascular events. These patients recovered after treatment, and none died during hospitalization. In the non-GIB patients, perforation occurred in one (0.6%) patient during endoscopic mucosal resection for a rectal carcinoid. No significant difference was observed among the four colonoscopy-related adverse events rates between LGIB and non-GIB patients, even after adjusting for confounding factors. In both the LGIB and non-GIB patients, there were no reports of death, intensive care, or surgery.

**Table 3:** Colonoscopy-related adverse events (n = 322).

	LGIB (n = 161)	Non-GIB (n = 161)	P	Crude OR (95% CI)	Adjusted OR* (95% CI)	P
Any	25 (15)	18 (11)	0.25	1.5 (0.76–2.94)	1.1 (0.51–2.55)	0.74
Hypotension	23 (14)	17 (11)	0.31	1.4 (0.72–2.83)	1.2 (0.51–2.59)	0.73
Perforation	0	1 (0.6)	1.00	NA	NA	NA
Cerebrocardiovascular events	2 (1)	0	0.50	NA	NA	NA
Sepsis	0	0	NA	NA	NA	NA

Values in parentheses are percentages. Hypotension was diagnosed as a systolic blood pressure <100 mmHg.

*The adjusted OR value took into account current alcohol use, antithrombotic drug use and the Charlson comorbidity index.

CI, confidence interval, GIB, gastrointestinal bleeding, LGIB, lower gastrointestinal bleeding, NA, not applicable, OR, odds ratio.

### Bowel preparation- and colonoscopy-related adverse events among subgroups of LGIB patients

Bowel preparation-related adverse events among various subgroups are shown in Table 4. No significant difference was observed among the five bowel preparation-related adverse event rates among subgroups of patients according to age >65, presence of comorbidities, or antithrombotic drug use. Colonoscopy-related adverse events among subgroups are shown in Table 5. No significant difference was observed among the four colonoscopy-related adverse events among subgroups.

**Table 4:** Preparation-related adverse events among LGIB patient subgroups (n = 161).

	Age ≥65 (n = 96)	Age <65 (n = 65)	P	Comorbidity index ≥2 (n = 66)	Comorbidity index <2 (n = 95)	P	With CHF (n = 12)	Without CHF (n = 149)	P	With CKD (n = 45)	Without CKD (n = 116)	P	Use of antithrombotic drugs (n = 51)	Non-use of antithrombotic drugs(n = 110)	P
Any	8 (8)	8 (12)	0.41	8 (12)	8 (8)	0.44	1 (8)	15 (10)	1.00	3 (6)	13 (11)	0.56	5 (10)	11 (10)	1.00
Hypotension	7 (7)	5 (8)	0.92	7 (11)	5 (5)	0.20	1 (8)	11 (7)	1.00	2 (4)	10 (9)	0.51	5 (10)	7 (6)	0.44
Volume overload	0	0	N/A	0	0	N/A	0	0	N/A	0	0	N/A	0	0	N/A
Vomiting	1 (1)	3 (4)	0.30	1 (2)	3 (3)	0.65	0	4 (3)	1.00	1 (2)	3 (3)	1.00	0	4 (4)	0.31
Aspiration pneumonia	0	0	N/A	0	0	N/A	0	0	N/A	0	0	N/A	0	0	N/A
Loss of consciousness	0	0	N/A	0	0	N/A	0	0	N/A	0	0	N/A	0	0	N/A

Values in parentheses are percentages. Hypotension was diagnosed as a systolic blood pressure <100 mmHg. The co-morbidity index was the Charlson comorbidity index. CHF, congestive heart failure, CKD, chronic kidney disease, LGIB, lower gastrointestinal bleeding, NA, not applicable.

**Table 5:** Colonoscopy-related adverse events among LGIB patient subgroups (n = 161).

	Age ≥65(n = 96)	Age <65(n = 65)	P	Comorbidity index ≥2 (n = 66)	Comorbidity index <2 (n = 95)	P	With CHF (n = 12)	Without CHF (n = 149)	P	With CKD(n = 45)	Without CKD (n = 116)	P	Use of antithrombotic drugs (n = 51)	Non-use of antithrombotic drugs (n = 110)	P
Any	11 (11)	14 (22)	0.08	11 (17)	14 (15)	0.74	3 (25)	22 (15)	0.40	7 (16)	18 (16)	1.00	8 (16)	17 (15)	1.00
Hypotension	11 (11)	12 (18)	0.21	9 (14)	14 (15)	0.84	3 (25)	20 (13)	0.38	7 (16)	16 (14)	0.77	7 (14)	16 (15)	0.89
Perforation	0	0	N/A	0	0	N/A	0	0	N/A	0	0	N/A	0	0	N/A
Cerebrocardiovascular event	0	2 (3)	0.16	2 (3)	0	0.17	0	2 (1)	1.00	0	2 (2)	1.00	1 (2)	1 (1)	0.54
Sepsis	0	0	N/A	0	0	N/A	0	0	N/A	0	0	N/A	0	0	N/A

Values in parentheses are percentages. Hypotension was diagnosed as a systolic blood pressure <100 mmHg. The co-morbidity index was the Charlson comorbidity index. CHF, congestive heart failure, CKD, chronic kidney disease, LGIB, lower gastrointestinal bleeding, NA, not applicable

## Discussion

We found that 16 (9%) LGIB patients experienced an adverse event during bowel preparation and, 25 (15%) experienced an adverse event during colonoscopy. None of the LGIB patients experienced volume overload, aspiration pneumonia, loss of consciousness, perforation or sepsis during colonoscopy, although 12 (7%) patients had hypotension and 4 (2%) vomited during bowel preparation, and 23 (14%) had hypotension and 2 (1%) experienced cerebrocardiovascular events during the colonoscopy procedure. However, these adverse event rates were not statistically different from those of elective colonoscopy among age- and gender- matched non-GIB cases. These adverse event rates were also low in LGIB patients grouped by advanced age, comorbidity presence, and antithrombotic drug use. These results suggest that colonoscopy in cases of acute LGIB did not increase adverse events.

A limited number of studies have identified adverse events following bowel preparation in LGIB patients. Zuckerman et al. summarized bowel preparation-related adverse events and reported cases of volume overload [4], and reported adverse events, such as hypotension, volume overload, aspiration pneumonia, and loss of consciousness, during elective colonoscopy [14]. Therefore, we evaluated these preparation-related adverse events.

Although some LGIB patients had a prior history of CHF 12 (7%) and CKD 45 (28%), none of them experienced volume overload. Previous studies have reported volume overload in only four cases among 13 reports [4]. These patients had hemodialysis and severe heart disease, and three underwent a high-volume saline purge, which can introduce volume overload. In contrast, PEG should pass through the bowel without being absorbed [15]. Thus, bowel preparation using PEG may be safe, even in LGIB patients with CHF or CKD.

We performed an intravenous infusion treatment in all hospitalized patients; however, 12 (7%) experienced hypotension during bowel preparation. Although no clinical reports on the adverse effects of hypotension during bowel preparation are available, physicians need to prepare for resuscitation using an intravenous infusion when patients become hemodynamically unstable.

Although 4 patients (2%) vomited in this study, none experienced aspiration pneumonia because medical staff provided adequate monitoring during bowel preparation. If vomiting occurs, medical staff can discontinue the preparation before aspiration pneumonia can develop. Previous studies reported that bowel preparation using a nasogastric tube increased the risk of aspiration pneumonia [16,17]. In this study, bowel preparation using a nasogastric tube was performed in only two LGIB patients and one non-GIB patients, which likely contributed to the decreased incidence of aspiration pneumonia.

We evaluated four colonoscopy-related adverse events identified in a previous study [4], and showed that none of the LGIB patients experienced perforation or sepsis, even though hypotension and cerebrocardiovascular events did occur. We performed resuscitation using intravenous infusion or transfusion during colonoscopy, but 23 (14%) LGIB patients had hypotension. Although no studies have reported on hypotension during colonoscopy in an acute LGIB setting, we suggest that blood loss may introduce hemodynamic changes in LGIB patients that result in hypotension. In contrast, it was reported that 5–7% of patients experienced hypotension in non-GIB elective colonoscopy [18]. In this study, 17 non-GIB patients (11%) had hypotension. It is likely that our population had increased comorbidities compared with the previous study, resulting in higher rates of hypotension.

Although no LGIB patients experienced a perforation in this study, such was reported in one patient in a previous study [19]. Loffeld et al. [20] reported that the presence of colonic diverticula was a risk factor for perforation in elective screening colonoscopy likely due to poor anatomical visualization [21]. Thus, caution should be taken in performing colonoscopy in acute LGIB, because diverticulosis is a major cause of LGIB [7,22]. In our study, two LGIB patients, who had a prior history of cerebral infarction and used antithrombotic drugs, experienced a cerebrocardiovascular event. However, both patients discontinued antithrombotic drug use because of GI bleeding. Witt et al. reported that discontinuation of warfarin therapy following GI bleeding was associated with an increased risk for thrombosis and death [23]. Thus, withdrawing warfarin therapy may contribute to a reduced incidence of re-bleeding, although it appears to increase the risk of cerebrocardiovascular events. However, the American Society for Gastrointestinal Endoscopy guidelines [2] have limited data available regarding the management of antithrombotic therapy in acute LGIB during hospitalization, and further large-scale studies are warranted to examine this issue.

In this study, no patients experienced sepsis, and among 13 previous reports, only one study reported sepsis in one patient [24]. That patient had an arterio-enteric fistula, which developed into sepsis due to Clostridium welchi. Colonoscopy may increase the risk of sepsis due to potential bacterial translocation. However, sepsis was a rarely seen adverse event.

LGIB has been significantly associated with a high comorbidity index [7]. However, there was no data available on the safety of colonoscopy in LGIB patients with a high comorbidity index. In this study, we analyzed the following LGIB subgroups: patients with advanced aged who had various comorbidities, patients with CHF and CKD who had a potential risk of volume overload, and patients using antithrombotic drugs with a potential risk of cerebrocardiovascular disease. There were no significant differences in bowel preparation- or colonoscopy-related adverse events among subgroups.

A strength of the present study was that we used a unified bowel preparation protocol and treatment algorithm. Furthermore, we were able to collect detailed patient background information, including comorbidities and medications. However, there were several limitations. First, we did not evaluate the endoscopic experience, which might affect adverse events. Second, the controls used may not reflect the ideal population, because there were some differences in comorbidities between cases and controls; thus, selection bias may have existed. Future studies may need to select age-, gender-, and comorbidity-matched control subjects. Third, our study was a retrospective analysis and 40 cases in LGIB were excluded because of inadequate adverse event information. The excluded patients represented a relatively large population, which might have decreased the statistical power for outcome analysis. Fourth, our study did not reach an adequate power to evaluate a difference of preparation-related adverse event rate between LGIB and non-GIB. Jensen et al. [25] reported an adverse event rate of 5% for preparation in LGIB patients, and we found a rate of 9%. A post hoc power calculation revealed a low power to detect a difference of preparation-related adverse event rate in LGIB vs. non-GIB (power = 0.61), which was not sufficient. Additional studies with larger sample size studies are needed. Finally, the timing of the colonoscopy after admission differed between LGIB and non-GIB subjects, which may have affected the risk of adverse events.

In conclusion, this study showed that the adverse event rates of bowel preparation and colonoscopy in acute LGIB patients were low and not statistically different from those in age- and gender-adjusted non-GIB cases. These adverse event rates were also low in elderly LGIB patients, as well as those with co-morbidities and antithrombotic drug use, suggesting that colonoscopy performed during acute LGIB did not increase adverse events.

## Supporting Information

S1 STROBE Checklist STROBE Checklist. STROBE STATEMENT checklist of items that should be included in reports of Observational Studies.
(DOC)

S1 Table Source of lower gastrointestinal bleeding (n = 161). (DOC)
