# Supplementary material for: Adverse Events during Bowel Preparation and Colonoscopy in Patients with Acute Lower Gastrointestinal Bleeding Compared with Elective Non-Gastrointestinal Bleeding
Source: PLoS One. 2015 Sep 14;10(9):e0138000. doi: 10.1371/journal.pone.0138000 (PMC4569066; doi:10.1371/journal.pone.0138000)
Supplement: S1 Table — (DOC) [file pone.0138000.s002.doc]

**Supplemental Table 1.** **Source of lower gastrointestinal bleeding (n = 161).**

| Diagnosis | No. of patients |
| --- | --- |
| Colonic diverticular bleeding | 78 (48) |
| Definite diagnosis | 19 (12) |
| Presumptive diagnosis | 59 (36) |
| Ischemic colitis | 29 (18) |
| Colorectal cancer | 5 (3) |
| Infectious colitis | 4 (2) |
| Angioectasia | 6 (4) |
| Rectal ulcer | 1 (1) |
| Inflammatory bowel disease | 8 (5) |
| Hemorrhoid | 5 (3) |
| Post polypectomy bleeding | 8 (5) |
| NSAIDs ulcer | 2 (1) |
| Non-specific ulcer | 2 (1) |
| Miscellaneous | 2 (1) |
| Definite small intestinal bleeding | 1 (1) |
| Presumptive small intestinal bleeding | 3 (2) |
| Unknown | 7 (5) |

Values in parenthesis denote percentages.

NSAIDs, non-steroidal anti-inflammatory drugs
